# Dr. Daniel Kimball Pearsons

**Published:** 1912-06

**Authors:** 


Dr. Daniel Kimball Pearsons, has recently died in Chicago at the age of 92. He gave up the use of tobacco a year ago, too late to prevent its deleterious influence in shortening life. He began the practice of medicine 70 years ago but as he gave away, in all five million dollars to philanthropy, it is scarcely necessary to state that he did not continue his active professional life but made a fortune buying and selling Illinois farm lands. One peculiarity regarding his gifts which does not seem to have struck any of the numerous press writers, is that his gifts were largely to educational institutions but that he gave nothing to the cause of medical training.

